# OsHLH61-OsbHLH96 influences rice defense to brown planthopper through regulating the pathogen-related genes

**DOI:** 10.1186/s12284-019-0267-0

**Published:** 2019-02-22

**Authors:** Meiling Wang, Dongyong Yang, Feilong Ma, Mulan Zhu, Zhenying Shi, Xuexia Miao

**Affiliations:** 1 Key Laboratory of Insect Developmental and Evolutionary Biology, Institute of Plant Physiology and Ecology, Shanghai Institutes for Biological Sciences, Chinese Academy of Sciences, Shanghai, 200032 China; 2 University of Chinese Academy of Sciences, Beijing, China; 3 Shanghai Chenshan Plant Science Research Center, Chinese Academy of Sciences/Shanghai Chenshan Botanical Garden, Shanghai, 201602 China

**Keywords:** *Oryza sativa*, bHLH, HLH, Transcription factor, Brown planthopper, *PR* gene

## Abstract

**Background:**

In plants, basic helix-loop-helix (bHLH) proteins form the largest transcription factor (TF) family. Among them, HLH proteins are a small group of atypical members that lack the basic domain, and form dimers with bHLH proteins. Although bHLH proteins have been proved to play important roles in plant development and physiology, the function of HLH proteins is rarely studied, not to mention in plant biotic resistance. Brown planthopper (BPH) is a kind of rice-specific insect that causes devastating yield losses each year.

**Results:**

In this study, we identified *OsHLH61* gene that encodes HLH protein. *OsHLH61* gene could be highly induced by BPH infestation. Furthermore, Methyl Jasmonic acid (Me-JA) and cis-12-oxo- phytodienoic acid (OPDA) induced expression of *OsHLH61*, while SA repressed it. We knocked down expression of *OsHLH61* by RNA interference (RNAi), the transgenic plants were susceptible to BPH infestation. RNA-seq analysis revealed that some pathogen-related (*PR*) genes in the Salicylic acid (SA) signaling pathway that mediate plant immunity were obviously down-regulated in the *OsHLH61* RNAi plants. Meanwhile, yeast two-hybrid assay and bimolecular luciferase complementation (BiLC) analysis identified bHLH096 to be an interacting factor of OsHLH61. Also, some *PR* genes were down-regulated in the *OsbHLH96* over expressing lines. Expression of *OsbHLH96* was inhibited. Besides, OsbHLH96 might interact with Jasmonate Zim-Domain3 (OsJAZ3).

**Conclusion:**

Altogether, we identified an OsHLH61–OsbHLH96 complex that might mediate defense to BPH through regulating *PR* genes. And OsHLH61–OsbHLH96 might be important in mediating SA and JA signaling crosstalk.

**Electronic supplementary material:**

The online version of this article (10.1186/s12284-019-0267-0) contains supplementary material, which is available to authorized users.

## Background

During their sessile growth, plants need to deal with various environmental stresses caused by biotic and abiotic factors. In thousands of years’ evolution, plants respond to these stresses through activation of series of responding molecules (Baniwal et al. 2004; Sunkar 2010). These defensing elements include TFs (Shi et al. 2018; Viana et al. 2018; Xiao et al. 2013), chaperone (Attallah et al. 2007), mitogen-activated protein kinase (Liang and Zhou, 2018), reactive oxygen species (ROS) (Miller et al. 2008), plant hormones (Bari and Jones, 2009; Peleg and Blumwald, 2011; Santino et al. 2013) and even sugar (Wingler and Roitsch, 2008).

bHLH proteins are the largest TF family in plants that function extensively in plant development and defensive response through crosstalk among different signaling pathways (Ezer et al. 2017; Kazan and Manners, 2013). The bHLH protein Myelocytomatosis protein 2 (MYC2) is involved in JA-regulated plant development (Dombrecht et al. 2007), root formation (Chen et al. 2011), insect resistance (Schweizer et al. 2013) and pathogen response (Kazan and Manners, 2013), and is becoming a master regulator of JA-mediated responses. Rice OsbHLH148 is involved in JA signaling by interacting with OsJAZ and mediates drought tolerance (Seo et al. 2011). However, HLH proteins are a small group of atypical members in the bHLH family that lack the basic domain and usually form dimers or multimers with bHLH proteins and inhibit their transcriptional activity (Carretero-Paulet et al. 2010; Li et al. 2006). In rice, positive regulator of grain length 2 (*PGL2*) regulates grain weight by influencing cell elongation and interacting with a bHLH protein Antagonist of Pgl1 (APG) (Heang and Sassa, 2012). In *Arabidopsis*, HLH proteins ATBS1-interacting factors (AIFs) and ILI1 binding bHLH (IBH1) respectively interact with paclobutrazol resistance1 (PREs) and activators for cell elongation 1 (ACE1) to form HLH-bHLH complex and mediate brassinosteroid (BR) signaling and cell elongation (Wang et al. 2009), and this regulating pathway is conserved in rice (Zhang et al. 2009). A recent study in rice indicates that a HLH protein BR upregulated 1-like (OsBUL1) regulates cell elongation by forming an OsBUL1 complex1 (Jang et al. 2017). Nevertheless, it is not clear whether HLH proteins function in stress response.

The brown planthopper (BPH) is a rice-specific herbivore, which causes severe yield losses each year in rice planting areas throughout Asia (Cheng et al. 2013; Flowers 2004). JA and SA mediated signaling pathways have been extensively identified in plant stress response against pathogen and insect (Berens et al. 2017). Generally, SA is proved to positively regulate rice defense to BPH (Yang and Zhang, 2016). Although the role of JA in BPH resistance is still controversy (Yang and Zhang, 2016), increasing evidence supports the negative role of JA in BPH resistance. For example, silencing of herbivore-induced rice type 2 13-lipoxygenase (*OsHI-LOX*) in JA synthesis is resistant to BPH (Zhou et al. 2009), and gain of function of 9-lipoxygenase gene (*Osr9-LOX1*) is favorable for the survival of the BPH larva (Zhou et al. 2014). The resistance genes *Bph14* and *Bph29* could both increase expression of genes in SA pathway and suppress genes in the JA pathway (Du et al. 2009; Wang et al. 2015). However, *Bph6* and *Bph9* could simultaneously activate SA and JA signaling pathway (Zhao et al. 2016; Guo et al. 2018), indicating that the specific role of JA in different background might also influence by the resistance genes. Nevertheless, the crosstalk between JA and SA in mediating BPH resistance is still unclear.

*PR* genes are key factors in the immune pathway and function as markers for systemic acquired resistance (SAR) response (Glazebrook 2005). Expression of several *PR1* genes is significantly induced by rice blast (Mitsuhara et al. 2008); and transgenic tobacco plants expressing high level of PR-la protein are resistant to pathogens (Alexander et al. 1993). Some PR1 proteins are proved to be antifungal (Niderman et al. 1995). Expression of some *PR* genes in SA signaling transduction is greatly influenced by the >non-expressor of pathogenesis-related genes 1(*OsNPR1*) in mediating resistance to bacterial and rice blast (Sugano et al. 2010). *Arabidopsis* monomer NPR1 can enter into the nucleus and interact with TGACG motif-binding factor (TGA), which directly regulates the transcription of some *PR* genes (Chern et al. 2014; Despres et al. 2000; Zhang et al. 2003). In rice, rTGA2.2 could interact with OsNPR1 (Chern et al. 2005), and rTGA 2.1 negatively regulates bacterial diseases and *PR10* gene (Fitzgerald et al. 2005). Transgenic rice lines carrying the *OsAOS2* gene under the control of a strong, pathogen-inducible promoter exhibit enhanced activation of some *PR* genes such as *PR1a*, *PR3*, and *PR5*, and increase resistance to rice blast (Mei et al. 2006). Also, some *PR* genes could be induced by BPH (Hu et al. 2017).

Previously, we revealed that over-expression of allene oxide cyclase (*AOC*) gene increased resistance to BPH in a JA-independent manner (Guo et al. 2014). To further reveal the downstream genes in *AOC-*mediated BPH resistance, we identified *OsHLH61*, which was up-regulated in *AOC* over-expressing plants. Function analysis revealed that *OsHLH61* positively regulated BPH resistance by influencing expression of *PR1*, *PR5* and *PR10* genes. OsbHLH96 was proved to be the interacting factor of OsHLH61, and OsJAZ3 might interact with OsbHLH96. Meanwhile, OsbHLH96 could regulate the expression of *PR* genes negatively. Therefore, we revealed the role of OsHLH61–OsbHLH96 complex in defense to BPH and added new points in SA and JA crosstalk in defending against BPH.

## Results

### Homologous comparison and phylogenetic analysis of the *OsHLH61* sequence

**Fig. 1:**
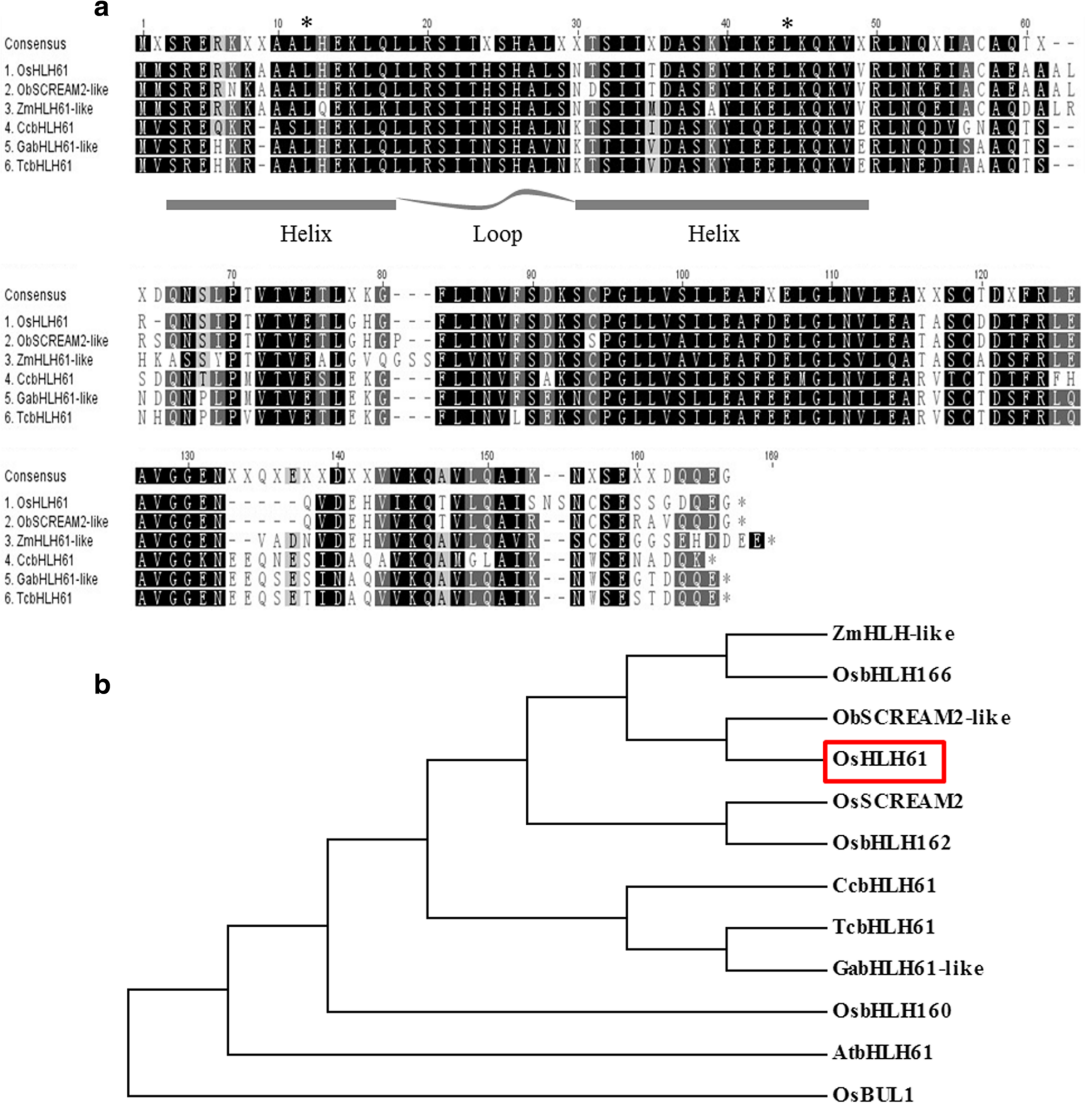
Sequence alignment and phylogenetic tree of the homologues genes of *OsHLH61*. **a** Sequence alignment of six HLH61 proteins from different species, the helix-loop-helix region was sketched, and asterisks indicated the conserved Leu12 and Leu44 positions. **b** Phylogenic tree of OsHLH61 and its homologues using the neighbor-joining method with 500 replications in Mega 6.0. OsHLH61 was indicated by red rectangular

We identified *OsHLH61* (Os07g0676600) gene in *AOC* over-expressing plants (Guo et al. 2014), and then cloned it. The coding region of *OsHLH61* gene contains 474 base pairs. The encoded protein OsHLH61 contains an atypical HLH domain in the 2–50 amino acids and belongs to group D (Atchley and Fitch, 1997), which lacks the basic domain, and so that could not directly bind to DNA (Li et al. 2006). Furthermore, members in group D could negatively regulate the bHLH proteins and repress their transcriptional activation (Sun et al. 1991). To further analyze *OsHLH61*, we selected some homologous genes in monocotyledonous plants *Oryza brachyantha* and *Zea mays,* and dicotyledonous plants *Cajanuy caja*, *Theobroma cacao* and *Gossyplum arboretum*, and performed sequence alignment. It was revealed that the HLH domains were highly conserved; and the α-helix in the HLH domain each had two conserved sites, Leu12 and Leu44 (Fig. 1a), which have been proved to be pivotal for dimerization (Brownlie et al. 1997; Carretero-Paulet et al. 2010). analysis using Neighbor-Joining, and revealed that OsHLH61 is nearest to the ObSCREAM2-like protein in wild rice (Fig. 1b).

### Expression profile of *OsHLH61* and subcellular localization of OsHLH61 protein

**Fig. 2:**
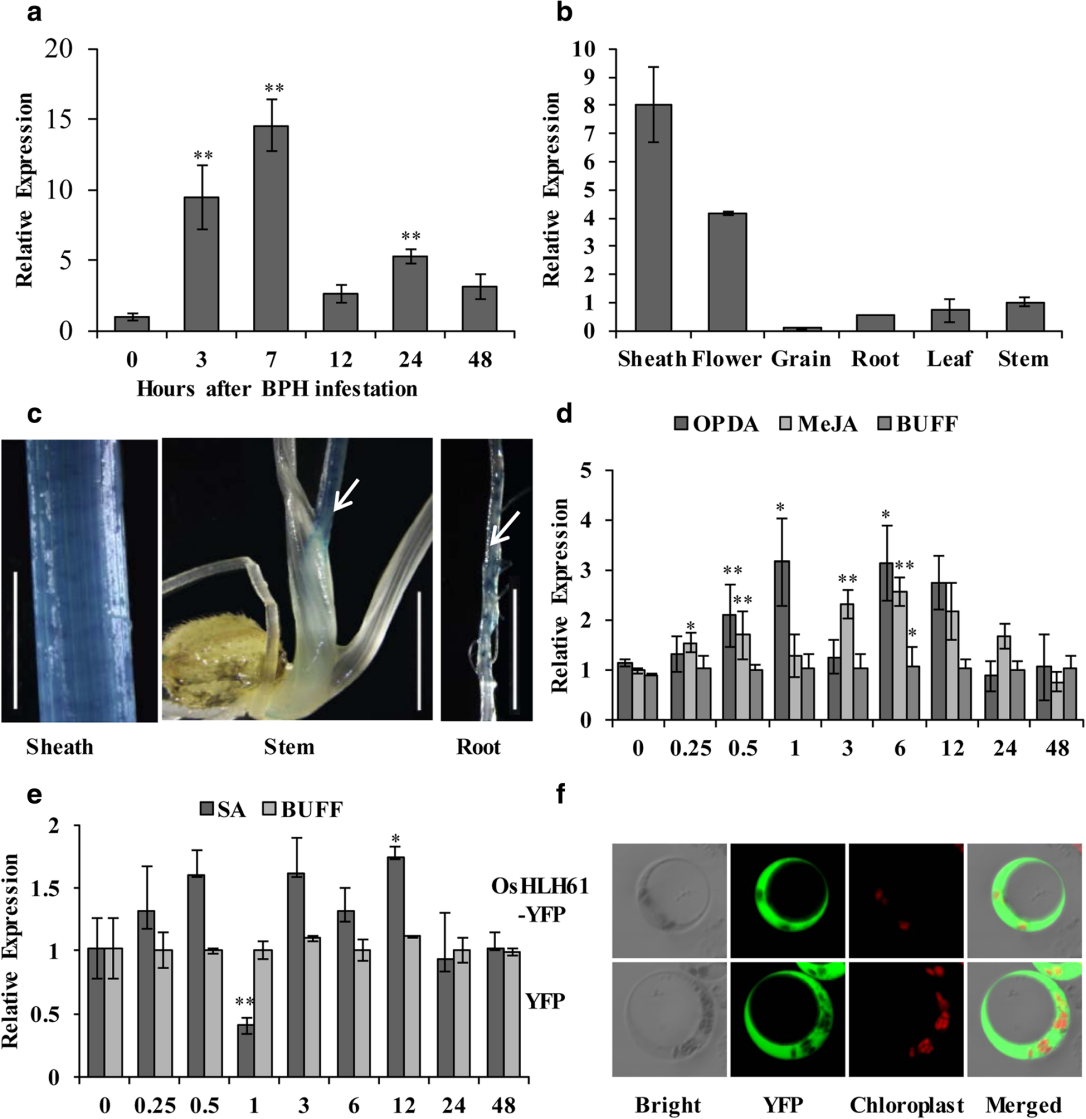
Expression profile of *OsHLH61* and subcellular localization of OsHLH61 protein. **a** Expression levels of *OsHLH61* transcripts after BPH infestation (*n* = 10). **b** Expression levels of *OsHLH61* transcripts in different tissues (*n* = 3). **c** Histochemical staining of the tissues in OsHLH61p::GUS transgenic plants, scale bar = 1 cm. **d** Expression levels of *OsHLH61* transcripts after OPDA and MeJA treatments (*n* = 3). **e** Expression levels of *OsHLH61* transcripts after SA treatment (*n* = 3). **f** Transient localization of OsHLH61-YFP in rice protoplast. Asterisks in (**a**), (**d**) and (**e**) represent significant differences determined by Student’s *t*-test at *P < 0.05 and **P < 0.01

To verify the response of *OsHLH61* to BPH, we checked expression of *OsHLH61* using a quantitative reverse transcriptase PCR (qRT–PCR) after BPH infestation, it was revealed that *OsHLH61* was induced as early as 3 h, and reached a peak at 7 h (Fig. 2a). So that *OsHLH61* can be induced by BPH infestation.

Next, we checked the expression of *OsHLH61* in different tissues, it was revealed that *OsHLH61* expressed highly in the leaf sheath (Fig. 2b), where BPH feeds. Furthermore, we constructed an OsHLH61p::GUS plants to monitor the tissues where OsHLH61 proteins expressed. In consistence, GUS signal was concentrated in the leaf sheath (Fig. 2c). Besides, GUS signal was also detected in the young stem, and the initiation sites of root hair (Fig. 2c).

Since expression of *OsHLH61* was influenced by *AOC*, which function in JA and OPDA biosynthesis (Guo et al. 2014), we further checked if *OsHLH61* was responsive to MeJA and OPDA, it was revealed that *OsHLH61* could be induced by both MeJA and OPDA (Fig. 2d). Meanwhile, under SA treatment, *OsHLH61* was down-regulated at 1 h (Fig. 2e).

To further study the molecular basis of the *OsHLH61* function, we checked the subcellular localization of the OsHLH61 protein. OsHLH61 localized ubiquitously in the protoplast, with the exception to the chloroplast (Fig. 2f), this was in consistence with that of OsBUL1 in rice (Jang et al. 2017). So that, HLH protein might be ubiquitously localized, different from bHLH protein, which are localized in the nucleus (Cui et al. 2016; Xu et al. 2014).

### Knockdown of *OsHLH61* rendered the plant more susceptible to BPH

**Fig. 3:**
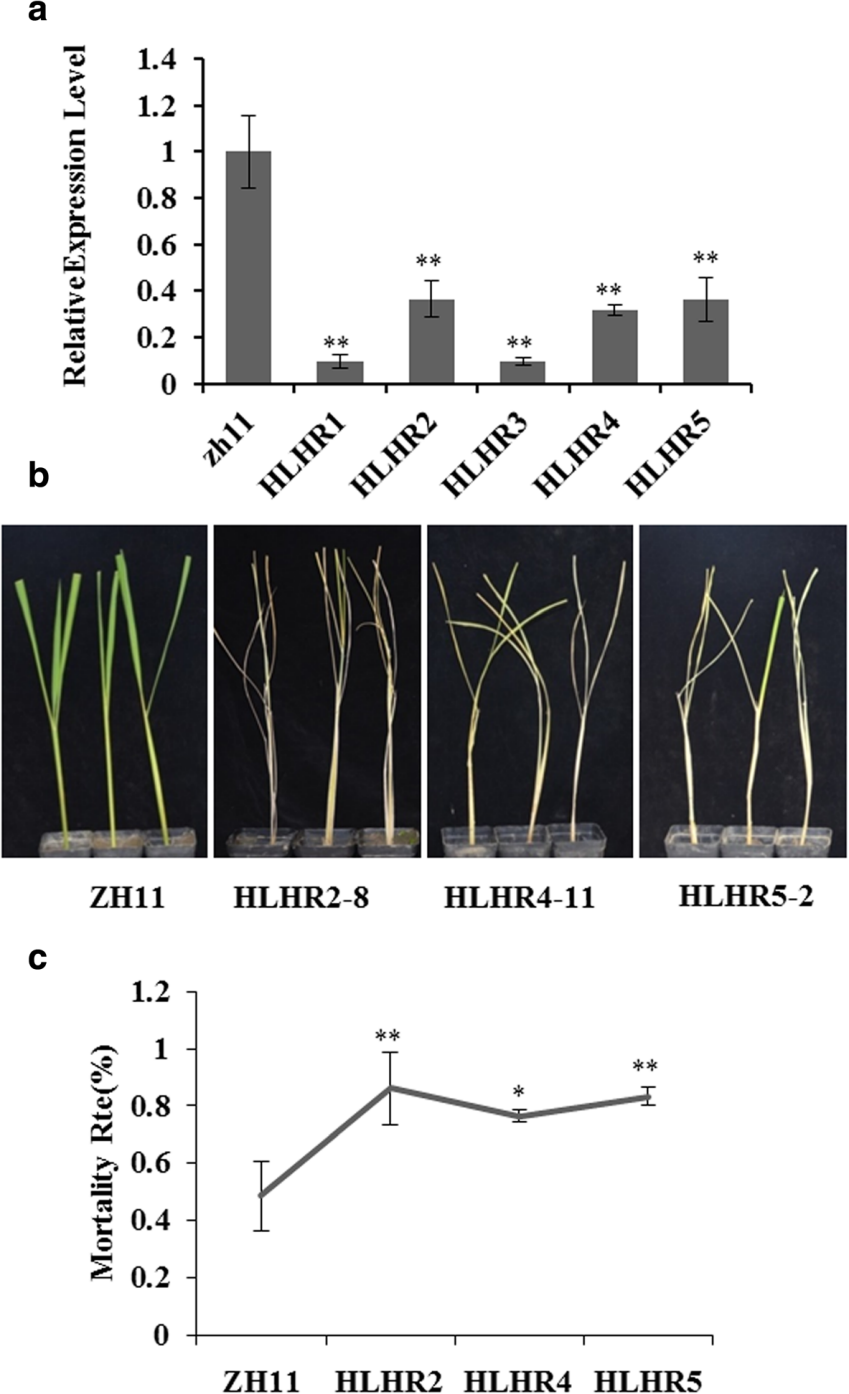
Functional characterization of *OsHLH61*. **a** Expression levels of *OsHLH61* transcripts in HLHR lines and WT (*n* = 3). **b** Phenotypes of HLHR lines after BPH infestation. **c** Statistic analysis of the survival rates of HLHR lines after BPH infestation (*n* = 30). Asterisks in (**a**) and (**c**) represent significant differences determined by Student’s *t*-test at *P < 0.05 and **P < 0.01

To investigate the genetic function of *OsHLH61*, we constructed RNAi plants (HLHR) of *OsHLH61* by transforming the RNAi plasmid into wild type (WT) ZH11. We chose 5 lines with obvious down-regulation of *OsHLH61* (Fig. 3a) for further analysis. Expression of *HLH61-like* (OsbHLH166, Os03g0338400), the homologous gene of *OsHLH61* in rice, was not influenced (Additional file 1: Figure S1a), indicating the specific down-regulation of *OsHLH61* in these HLHR plants. Then we performed individual analysis and revealed that the HLHR plants were more susceptible to BPH infestation (Fig. 3b). In consistence, the mortality of the HLHR plants was much higher than the WT after BPH infestation (Fig. 3c).

Besides, knock down of *OsHLH61* influenced rice development. The leaves of the HLHR plants curled adaxially (Additional file 1: Figure S1b) and the tiller number increased (Additional file 1: Figure S1c). In addition, the fertility of the HLHR plants was reduced (Additional file 1: Figure S1 d, e).

### OsHLH61 interacted with OsbHLH96

**Fig. 4:**
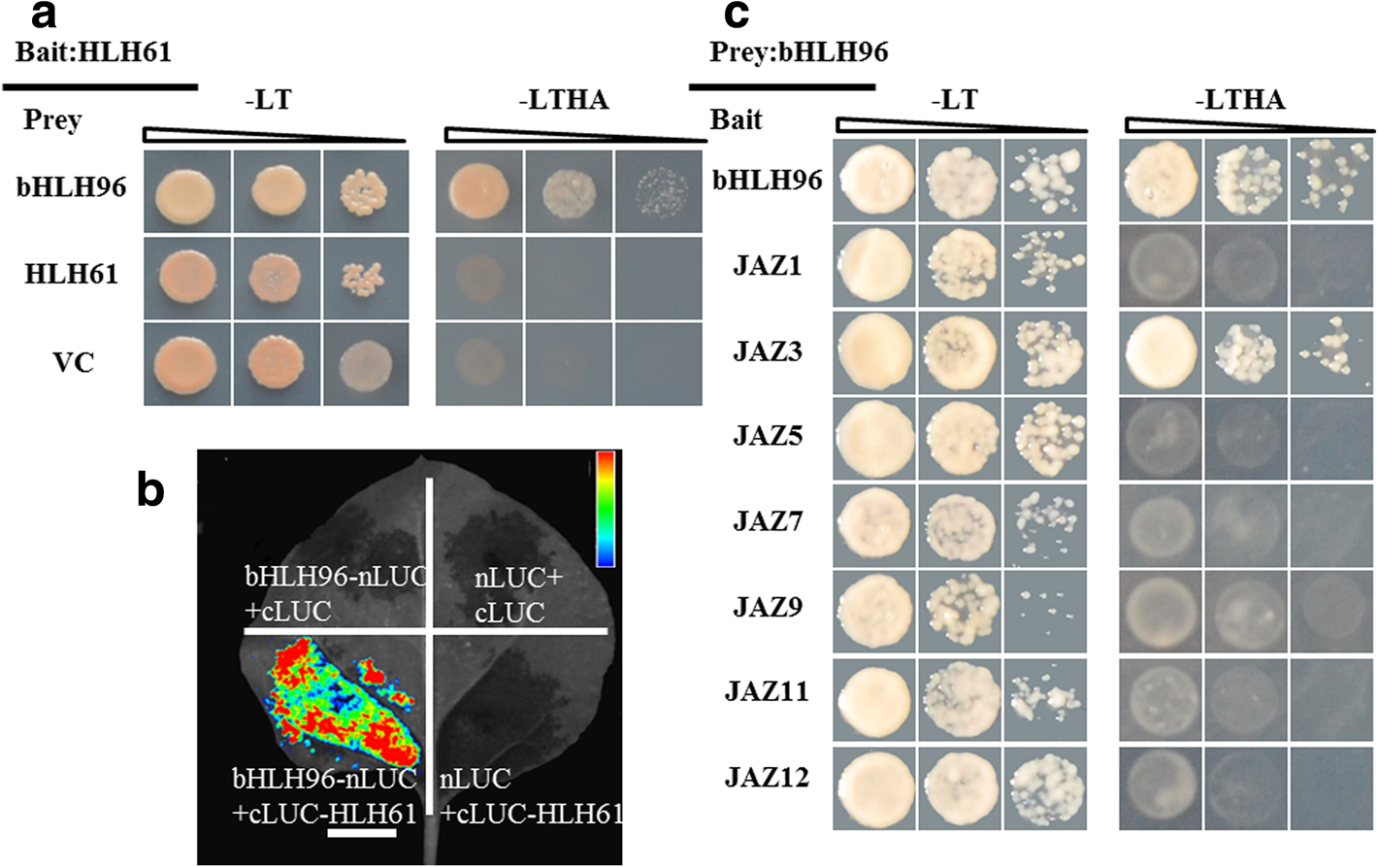
Interaction analysis of OsHLH61 and OsbHLH96, and OsbHLH96 and OsJAZ3. **a** Yeast two-hybrid analysis of OsHLH61 and bHLH96; **b** BiLC assay of OsHLH61 and OsbHLH96 in tobacco. **c** Yeast two-hybrid analysis of OsJAZs and OsbHLH96. -LT, −LTHA indicated SD medium without Leu and Trp amino acids, and Leu, Trp, His and Ade amino acids respectively

Since OsHLH61 was an atypical bHLH protein that needs to form HLH-bHLH complex in functioning, we tried to search the possible interacting bHLH protein of OsHLH61. There are 177 bHLH proetins in rice, with 26 atypical HLHs and 151 typical bHLHs (Carretero-Paulet et al. 2010; Li et al. 2006). We screened a yeast library and identified one bHLH protein, OsbHLH96, to interact with OsHLH61 in yeast two-hybrid analysis (Fig. 4a). BILC assay verified the interaction between OsHLH61 and OsbHLH96 (Fig. 4b). We found that OsHLH61 could not form homodimers in yeast two-hybrid test (Fig. 4a), but OsbHLH96 could (Fig. 4c). So that OsbHLH96 is the interacting protein of OsHLH61.

It is reported that AtMYC2 (Thireault et al. 2015) and OsbHLH148 (Seo et al., 2011) can respectively interact with JAZs in functioning. We wondered if OsbHLH96 could interact with OsJAZs, so that we checked OsJAZ1, OsJAZ3, OsJAZ5, OsJAZ7, OsJAZ9, OsJAZ11 and OsJAZ12, and revealed that only OsJAZ3 could interact with OsbHLH96 in yeast two-hybrid analysis (Fig. 4c).

### Some *PRs* were significantly down-regulated in *OsHLH61* RNAi plants

**Fig. 5:**
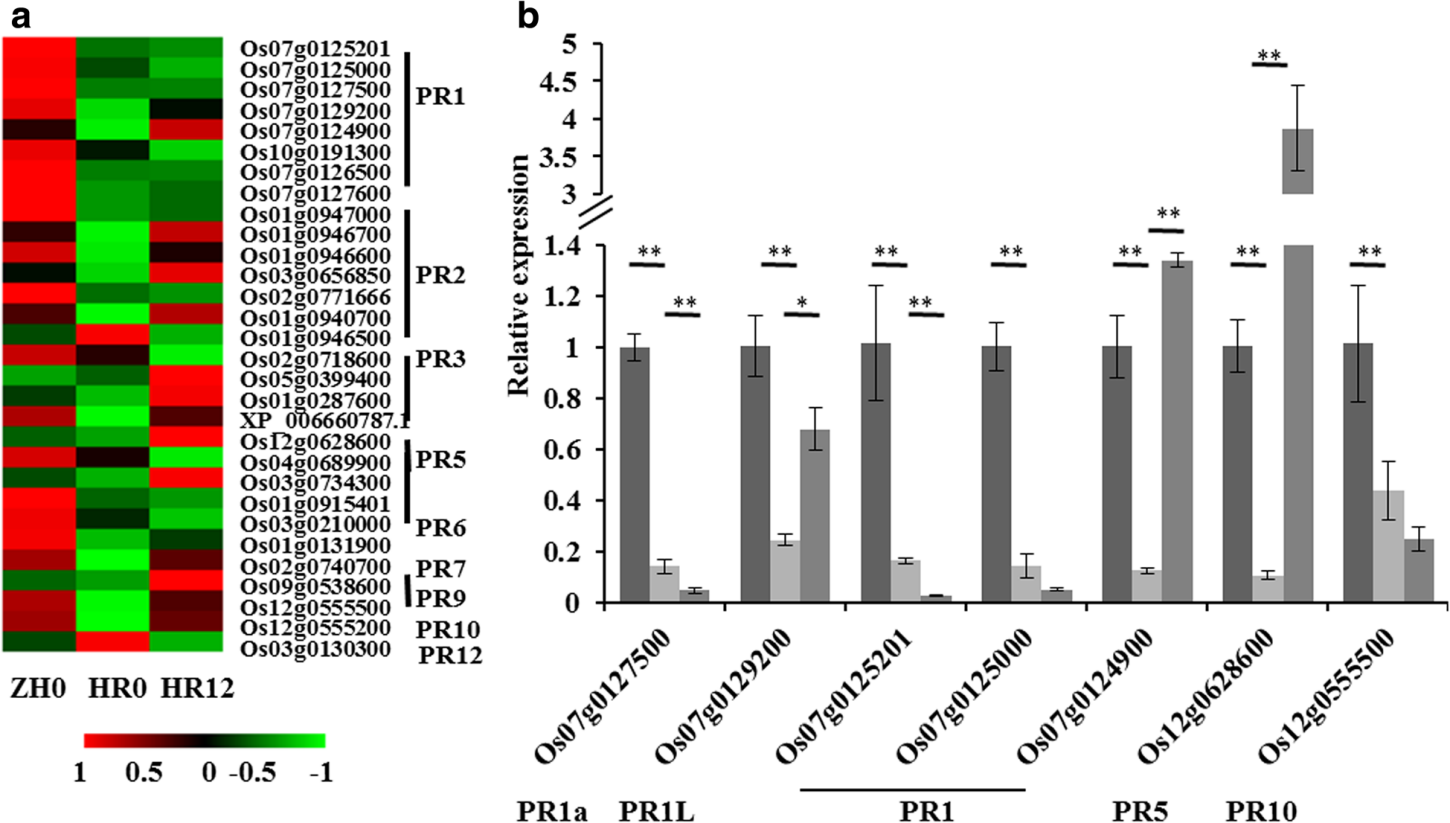
Expression analysis of *PR* genes in HLHR plants. **a** Expression levels of *PR* transcripts in WT and HLHR plants revealed by the RNA-seq analysis. **b** qRT-PCR analysis of some *PR* genes in the HLHR-4 plants before and after BPH infestation, and in the WT (*n* = 3). Asterisks in (**b**) represent significant differences determined by Student’s *t*-test at *P < 0.05 and **P < 0.01

To analyze the genes involved in *OsHLH61* functioning, we carried out a RNA-seq analysis of the HLHR-4 plants before and after BPH infestation. Three kind of samples, WT ZH11, HLHR-4 without BPH infestation, and HLHR-4 after BPH infestation for 12 h (named as ZH0, HR0 and HR12 respectively), were used for analysis. We found that most *PR* genes were down-regulated in HR0 compared with in ZH0, so that data of *PR* genes were extracted and displayed in heat map (Fig. 5a), it was revealed that most *PR* genes in HR0 were down-regulated. Because the expression of *PR1a* (Os07g0129200), *PR5* (Os04g0689900) and *PR10a* (Os12g0555500) were influenced by BPH feeding (Hu et al. 2017), we further checked the expression of *PR1a*, *PR5*, *PR10a*, *PR1-like* (*PR1L*, Os07g0127500) and some *PR1* (Os07g0125201, Os07g0125000, Os07g0124900) by qRT-PCR. It was revealed that all of them were down-regulated in the HR0 plants (Fig. 5b). Besides, expression of the *OsHLH61-like* was not influenced in RNA-seq analysis, further indicating that *OsHLH61* was specifically down-regulated in the HLHR plants (data not shown).

### Over-expression of *OsbHLH96* down-regulated expression of some *PR* genes

**Fig. 6:**
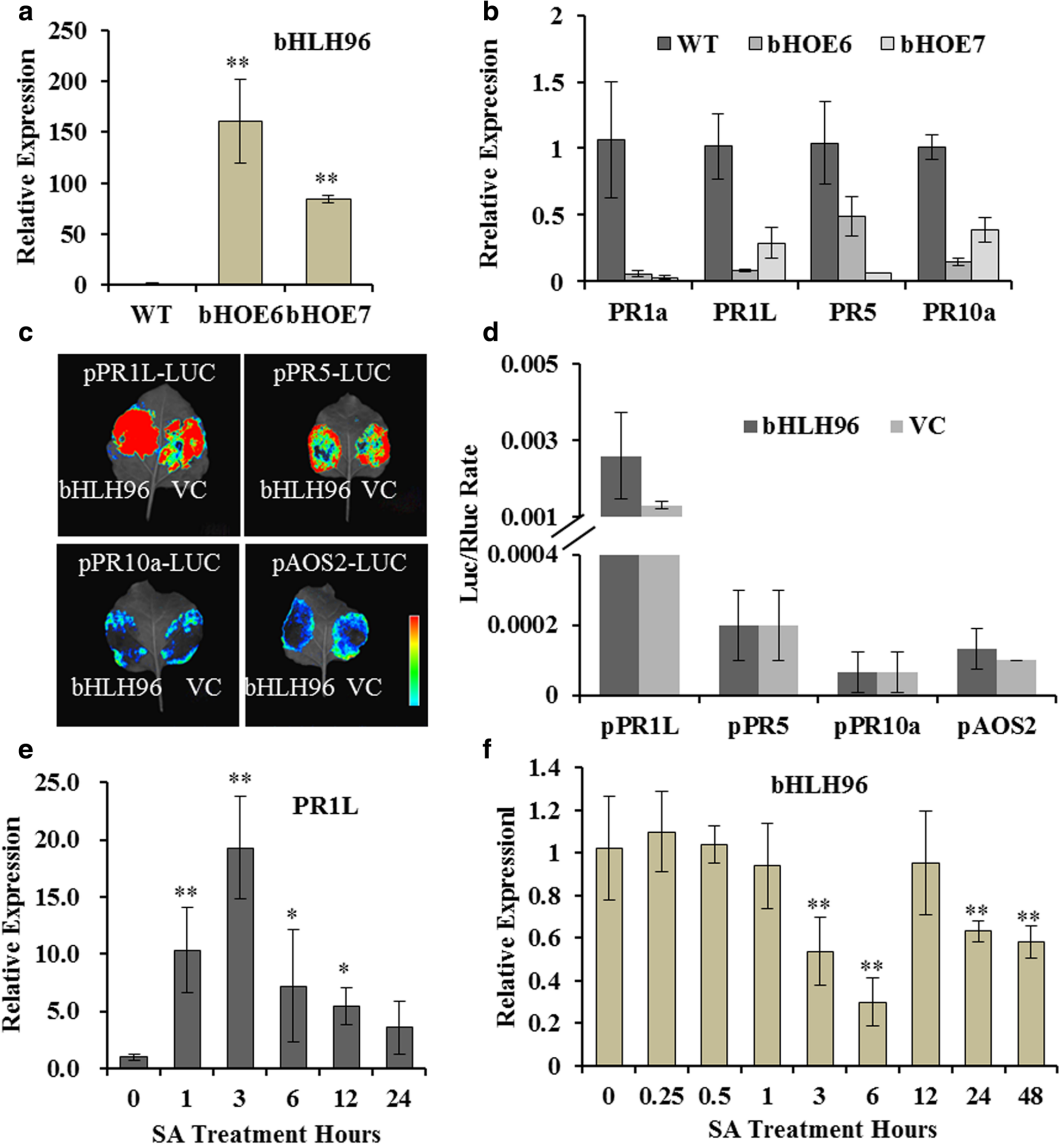
OsbHLH96 regulated the transcriptional levels of some *PR* genes. **a** qRT-PCR analysis of the *OsbHLH96* transcripts in the *OsbHLH96* over-expressing lines (bHOE) and the WT. **b** Expression levels of some *PR* gene transcripts in the *OsbHLH96* over-expressing lines. **c** Dual-Luc assay of the OsbHLH96 and the promoters of *PR* genes. P, promoter, VC, Vector control. **d** Quantitative test of Luc/Rluc ratio. **e** Expression levels of the *PR1L* transcripts after SA treatment; **f** Expression levels *OsbHLH96* transcripts after SA treatment. Asterisks represent significant differences determined by Student’s t-test at *P < 0.05 and **P < 0.01, *n* = 3

Now that OsbHLH96 is the interacting protein of OsHLH61, we want to know if OsbHLH96 could regulate expression of *PR* genes. We constructed the over-expression lines of *OsbHLH96* (bHOE) and selected two positive lines for further analysis (Fig. 6a). Through qRT-PCR analysis, it was revealed that *PR1a*, *PR1L*, *PR5* and *PR10a* genes were down-regulated in *OsbHLH96* over-expressing lines (Fig. 6b). Now that expression of *PR* genes was influenced by *OsbHLH96*, we further analyzed if there are any E-box motifs for bHLH protein binding in the promoters of the *PR* genes (Goossens et al. 2016). The promoters of *PR1a*, *PR5*, *PR10a* and *AOS2* (Os03g0225900) have some E-box motifs (http://plantpan2.itps.ncku.edu.tw). Next, we performed Dual-Luc assay to determine whether OsbHLH96 could directly regulate *PR* genes. Neither the luciferase signals (Fig. 6c) nor the LUC/RLUC ratios (Fig. 6d) support a direct regulation. *PR* genes can be induced by SA (Agrawal et al., 2000), we chose *PR1L* to determine its expression under SA treatment. It was revealed that *PR1L* was induced by SA (Fig. 6e). However, *OsbHLH96* was repressed by SA treatment (Fig. 6f).

## Discussion

In this study, we revealed that down-regulation of *OsHLH61* showed significant sensitivity to BPH, and proved the function of HLH–bHLH heterodimer in BPH response. We showed that OsHLH61 form bHLH–HLH heterodimer with OsbHLH96 (Fig. 4a, b). In both *OsHLH61* RNAi and OsbHLH96 over-expressing plants, *PR* genes were down-regulated (Fig. 5a, b; Fig. 6b). *PR1a* gene can be induced by small brown planthopper indigestion (Hao et al. 2011), and *PR1b* has been reported to be induced by brown planthopper indigestion (Hu et al. 2017). Some PR1 proteins are proved to be antifungal (Niderman et al. 1995). Our study indicated that down regulation of *PR* genes in *OsHLH61* RNAi plants might account for their sensitivity to BPH (Fig. 3b).

*PR* genes are considered as markers for plant resistance (Glazebrook, 2005, Liu et al., 2018, Liu et al., 2013). Some *PR* genes were down-regulated in *OsHLH61* RNAi plants (Fig. 5). *OsHLH61* was induced by JA, OPDA, but repressed by SA (Fig. 2d, e), and up-regulated in *AOC* and *OPR3* over-expressing lines (Guo et al., 2014), while the transcriptional level of *AOC* or *OPR3* was unchanged in *OsHLH61* RNAi plants (data not shown), demonstrating that *OsHLH61* located downstream of both *AOC* and *OPR3* in JA synthetic pathway. *OsbHLH96* was inhibited by SA (Fig. 6g), while *PR* genes were induced by SA (Fig. 6f), together with the down-regulation of *PR* genes in the *OsbHLH96* over-expressing lines (Fig. 6b), indicating *OsbHLH96* to be a negative regulator of SA signaling pathway in mediating resistance. In this study, we proved that OsHLH61 could interact with OsbHLH96 (Fig. 4a, b). And OsJAZ3, a JA pathway repressor (Chini et al., 2007), might interact with OsbHLH96 (Fig. 4d). Therefore, the OsHLH61–OsbHLH96 complex might mediate the crosstalk between SA and JA in regulating BPH resistance.

Furthermore, OsJAZ3 interacts with OsCOI1a (Os01g0853400) in the presence of 120 μM coronatine (Seo et al., 2011). *OsCOI1a*-silenced plants are more sensitive to chewing insects (Ye et al., 2012), Meanwhile, *OsCOI1a* influence the crosstalk between JA and GA (Yang et al., 2012). So that OsHLH61-OsbHLH96-OsJAZ3 might form multimers in stress response and plant hormone crosstalk.

Some TFs regulate *PR* genes directly (Zhang et al., 2003; Chern et al., 2014). Although expression of some *PR* genes were down-regulated in the *OsHLH61* RNAi plants and the *OsbHLH96* over-expressing lines (Fig. 5; Fig. 6f), direct regulation of OsbHLH96 to the *PR* genes was not detected (Fig. 6c, d). There might be other TFs to regulate *PR* genes instead. In the *OsHLH61* RNAi plants, expressions of more than 30 TF-encoding genes were influenced (Additional file 2: Table S2). These TFs might be powerful candidates for direct regulation of *PR* gene.

## Conclusion

In this study, *OsHLH61* RNAi plants were more susceptible to BPH than WT. OsHLH61 and OsbHLH96 can form heterodimer in functioning, and regulate the expression of *PR* genes positively and negatively respectively, their antagonism in regulating *PR* genes might be important to the understanding of the crosstalk between SA and JA signaling in mediating BPH resistance.

## Materials and methods

### Plant and insect

The WT rice variety is ZH11 (*Oryza sativa L. subsp*. *japonica* cv. Zhonghua No.11). Rice plants were grown in a greenhouse at 28 ± 2 °C with a 12-h light/12-h dark cycle, and 70%–80% relative humidity. The BPH population was originally obtained from rice fields in Songjiang, Shanghai, China, and maintained on plants in a climate-controlled room at 26 ± 2 °C, 12-h light/12-h dark cycle and 80% relative humidity, or in the field under natural condition.

### BPH performance measurements

Individual plant test was carried out at seedling stage using at least six replicates of each cultivar or line as previously described (Wang et al. 2012; Zhao et al. 2016). Each seedling about 3-week-stage was put under a plastic cage (diameter 4 cm, height 8 cm, with a breather window) infested with 12–18 s-instar BPH nymphs. Plant damage levels were observed daily, and 6–9 days later, the plants were scored as susceptible (dead) or resistant (alive).

### Constructs

To construct the OsHLH61-RNAi plasmid, a 273-bp gene-specific fragment of the OsHLH61 coding sequence was amplified and cloned into PTCK303 vector in sense orientation by *Bam*HI and *Kpn*I, and in antisense orientation by *Sac*I and *Spe*I.

To construct *OsbHLH96* over-expression plasmid, the full-length *OsbHLH96* was cloned into the *Bam*HI and *Kpn*I sites of pCambia1301-35SNOS vector, and got bHLH96OE plasmid.

Constructs used for rice protoplast transfection was generated with pA7-YFP, *OsHLH61* cDNA sequence was cloned into the vector with pA7-HLH61-YFPF and.

pA7-HLH61-YFPR primers. For construction of OsHLH61p::GUS plasmid, a 2.27 kb promoter of *OsHLH61* was cloned into p1300GUSNOS vector.

All the plasmids for genetic transformation were transformed into ZH11 using *Agrobacterium*-mediated method (Hiei et al., 1994). GUS activities were histochemically detected as described (Jefferson 1989).

For yeast two-hybrid analysis, the coding sequences of *OsHLH61* and *OsbHLH96* were cloned into pGADT7 and pGBKT7 vectors, the coding sequences of *OsJAZ1*, *OsJAZ3*, *OsJAZ5*, *OsJAZ7*, *OsJAZ9*, *OsJAZ11* and *OsJAZ12* were cloned into the pGADT7 vector. For BiLC analysis, the coding sequences of OsbHLH96 and OsHLH61 were cloned in-frame into the *Kpn*I and *Sal*I sites of 771-nluc and cluc-772 vector respectively.

For Dual-Luc analysis, the 2 kb promotor of *OsPR1a*, *PR1L*, *OsPR5*, *OsPR10a* and *OsAOS2* were cloned into the *Kpn*I and *Bam*HI sites of pGreenII vector. The bHLH96OE plasmid and these plasmids were used for Dual-Luc analysis analyses (see the following).

### Rice protoplast transformation

Rice protoplast transformation was performed by using polyethylene glycol -mediated transfections as described (Zhang et al. 2011). The YFP fluorescence signals for each combination were detected using an inverted confocal microscope (Olympus FV1000) 16 h after incubation. YFP fluorescent and chlorophyll auto-fluorescent signals were imaged at 514 nm, 527–532 nm and 650–798 nm respectively.

### Plant treatments

For BPH treatment, plants about 2-week-old were infested with second-instar BPH nymphs after starvation for 2 h at a rate of 5 insects per seedling, and stem were collected after 0, 3, 6, 12, 24 and 48 h. For phytohormone treatment, rice plants were sprayed with 4 mL of MeJA (400 μM), OPDA (100 μM), or SA (500 μM) solution in Dimethyl sulfoxide (DMSO), which was sprayed as control. Plant samples at 0, 0.5, 1.5, 3, 6, 12, 24 and 48 h were collected and stored at − 80 °C before RNA extraction.

### Quantitative real-time PCR (qRT–PCR) analysis

Stem of 14-day-old WT plants were used to examine transcript levels of target genes before/after plants were infested with BPH. Three independent biological samples were used. Total RNA was isolated by using Trizo (Thermo). 1 μg of total RNA was reverse-transcribed using the First Strand cDNA Synthesis Kit (Toyobo), according to the manufacturer’s protocol. The qRT–PCR was performed with the SYBR Green Real-time PCR Master Mix Kit (Toyobo). *Ubiquitin* (Os03g0131300) was used as an internal standard to normalize cDNA concentrations.

### Data analysis

Data differences in different lines or treatments were determined by analyzing variance followed by Student’s *t*-test. All tests were carried out with GraphPad Prizm (https://www.graphpad.com/scientific-software/prism/).

### RNA-seq and analysis

Seedlings of non-infected HLHR4 and ZH11 plants, and of BPH infected HLHR4 plants were collected and RNAs extracted. Library was constructed, and sequencing was performed on a BGISEQ-500 and analysis was carried out under the help of Beijing Genomic Institution (www.genomics.org.cn, BGI, Shenzhen, China).

### Tree building

A phylogenetic tree was constructed using MEGA 6.0 (http://www.megasoftware.net/index.html) and the NJ method with the following parameters, Poisson correction, pairwise deletion, and bootstrap (500 replicates; random seed).

### Yeast two hybrid screening

The yeast two-hybrid screening was carried out as described by Clontech (https://www.takarabio.com/products/protein-research/two-hybrid-and-one-hybrid-systems/yeast-two-hybrid-system/matchmaker-gold-yeast-two-hybrid-system). The vector pGBKT7-OsHLH61 was transformed into AH109 instead of Y2HGOLD. The mated culture was plated on the medium lacking leucine, tryptophan, histidine and adenine QDO (SD-LTHA) agar plates, the culture medium plates were incubated for 3–5 d, then put the colonies into new plates QDO for 3–5 d, yeast colony PCR were carried out and the PCR products sequenced.

Yeast two-hybrid assay was carried out using the lithium acetate/single-stranded carrier DNA/PEG method (Gietz and Schiestl, 2007).

### Bimolecular luciferase complementation (BiLC) assay and dual-luciferase (dual-Luc) reporter assay

BiLC assay was carried out as described (Liu et al. 2018). OsbHLH96 was fused to nLUC, OsHLH61 was fused to cLUC. Both constructs were transformed into *Agrobacterium* strain GV3101. Overnight cultures were collected by centrifugation, re-suspended in MES buffer (10 mM MES pH 5.6, 10 mM MgCl2, 150 mM acetosyringone) to an OD600 of 1.0, mixed, nLUC and cLUC, bHLH96-nLUC and cLUC, nLUC and cLUC-HLH61 were used as control, and incubated at room temperature for 2–3 h. The *Agrobacteria* suspension was drawn into a 10 mL syringe (without the needle) and press carefully by hand into healthy leaves of 3-week-old *N. benthamiana* plants. The plants were left under continuous white light for 2 d after infiltration. 1 mM luciferin was infiltrated before the LUC signal was photographed with a cool CCD camera (Tanon 5200).

For Dual-Luc assay, OsbHLH96 and OsHLH61 were cloned into p1301-35SNOS vector, and the promotors of *PR* genes were cloned into pGreenII0800. Constructs for tests were then transformed into *Agrobacterium* strain GV3101 containing the pSoup-P19 plasmid (Hellens et al. 2005). LUC/RLUC ratio was measured using the Dual-Luciferase® Reporter Assay System kit (Promega).

The primer sequences used in this study were listed in Additional file 3: Table S2.

### Accession numbers

Sequences in this study can be found in the National Center for Biotechnology Information (NCBI) *OsHLH61* (Os07g0676600); *OsbHLH96*(Os03g0188400); *OsJAZ3*(Os08g0428400); *OsPR1a* (Os07g0129200); *PR1L* (Os07g0127500); *OsPR5* (Os04g0689900); *OsPR10a*(Os12g0555500); *OsAOS2* (Os03g0225900); *OsJAZ1*(Os04g0653000); *OsJAZ5*(Os04g0395800); *OsJAZ7*(Os07g0615200); *OsJAZ9*(Os03g0180800);*OsJAZ11*(Os03g0180900); *OsJAZ12*(Os10g0392400); *Ubiquitin*(Os03g0131300).

## Additional files

Additional file 1: **Figure S1.** Other phenotypes of the HLHR plants. (DOCX 626 kb)

Additional file 2: **Table S1.** List of TF genes influenced by *OsHLH61*. (XLSX 11 kb)

Additional file 3: **Table S2.** Primer sequences used in this study. (XLSX 11 kb)

## Abbreviations

OsAOS2: Allene Oxide Synthase 2
OsHLH61: Helix-loop-helix 61
PRs: Pathogen-related genes
qRT-PCR: Quantitative real-time PCR
