# Supplementary material for: OsHLH61-OsbHLH96 influences rice defense to brown planthopper through regulating the pathogen-related genes
Source: Rice (N Y). 2019 Feb 22;12:9. doi: 10.1186/s12284-019-0267-0 (PMC6386760; doi:10.1186/s12284-019-0267-0)
Supplement: Supplementary file 1 — Figure S1. Other phenotypes of the HLHR plants. (DOCX 626 kb) [file 12284_2019_267_MOESM1_ESM.docx]

**Additional file 1: Figure S1 Other phenotypes of HLHR plants**

**
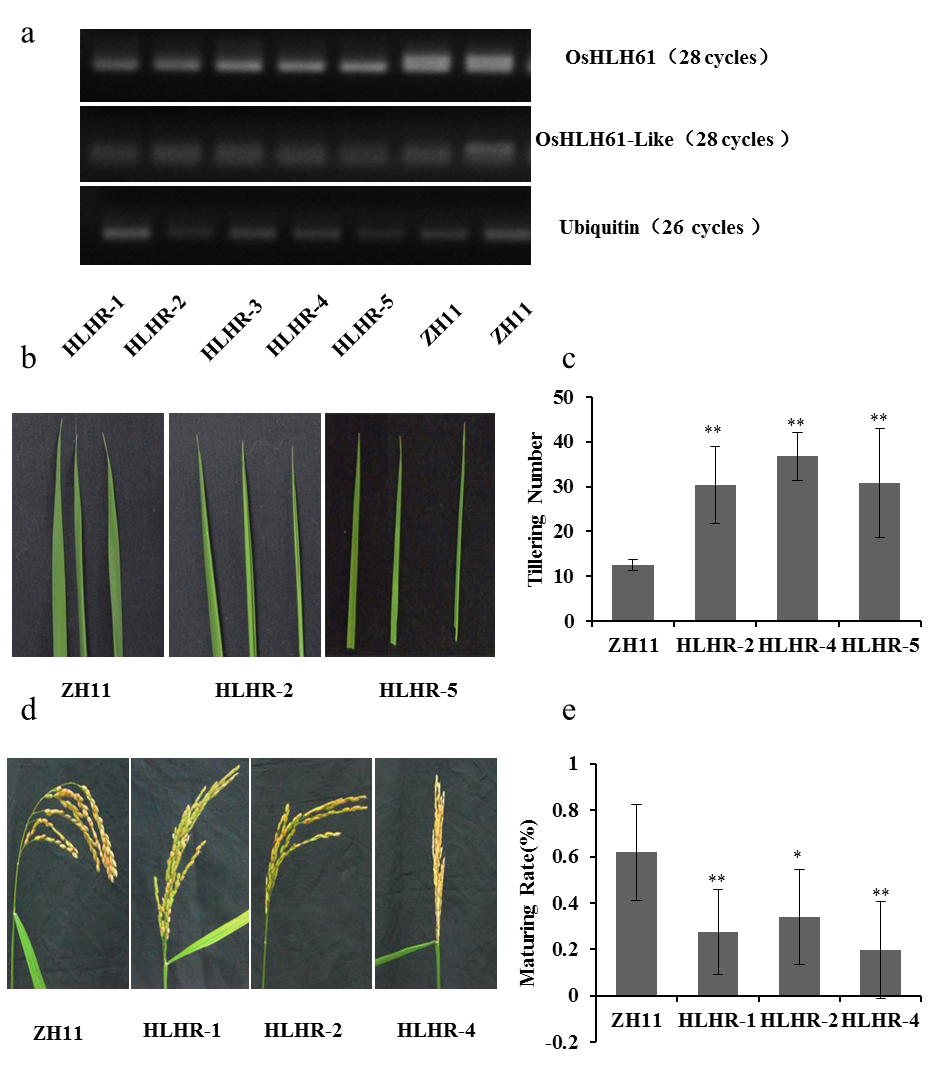
**

**Figure S1 Other phenotypes of HLHR plants.**

**a** Expression of *OsHLH61 and OsHLH61-like* in HLHR plants byq RT-PCR. **b** Leaf phenotype of HLHR plants. **c** Statistical analysis of the tiller number of HLHR plants in the field (n = 10). **d** Phenotype of mature panicles in the field. **e** Statistical analysis of the maturing rate of the HLHR lines in the field. Asterisks in (c) and (e) represent significant differences determined by Student’s t-test at *P<0.05 and **P<0.01.
